# Dynamic road section risk identification model based on connected in-vehicle data

**DOI:** 10.1371/journal.pone.0333623

**Published:** 2025-10-06

**Authors:** Yongjian Zhang, Ge Yang, Tian Xie

**Affiliations:** 1 School of Civil Engineering, Central South University of Forestry & Technology, Changsha, China; 2 Hunan Expressway Group Co., Ltd, Changsha, China; 3 CCSHCC Traffic Engineering Co. Ltd, Wuhan, China; 4 Intelligent Transportation System Research Center, Wuhan University of Technology, Wuhan, China; Nanjing Forestry University, CHINA

## Abstract

To dynamically identify road section risks, the research team designed an onboard unit to collect various dynamic driving behavior data when the Advanced Driver Assistance Systems (ADAS) are activated. To do this, we divided the roads into three categories (urban road, expressway, freeway) and established separate BN models to analyze the relationship between driving behavior and road section risk. These models were constructed based on natural driving data from 10,000 km, collected from vehicles equipped with ADAS. For road segment division, fixed-length intervals were used for freeways and urban expressways, while segments on urban roads were defined as the stretches between adjacent intersections. Using braking deceleration and time to collision, we identified near-crash events and classified them into high, medium, and low severity levels using the DBSCAN clustering algorithm. These near-crash events were then matched to the corresponding road section, assigning different weights based on their severity levels to evaluate the risk level of each segment. Additionally, driving behavior data, including velocity, lateral acceleration, longitudinal acceleration, yaw rate, accelerator position, steering angle, and steering angle velocity, were matched to the road segments. Finally, using the Netica software, Bayesian network models were constructed separately for urban roads, expressways, and freeways to identify driving risks at road segments. The models exhibited high sensitivity to observed nodes.

## 1. Introduction

How to identify risks, reduce traffic accidents, and improve traffic safety are key priorities in traffic management. At present, most risk identification models rely on historical accident data for verification. The common approach is to employ cluster analysis in accident data mining [1]. This involves clustering accident black spots based on the accident rate index statistics of each kilometer of road section, thus revealing the distribution patterns of accidents within a specific time frame. For instance, Krsto et al. [2] utilized both subjective methods, such as field research and expert interviews, and objective methods, utilizing historical and spatial distribution data of road accidents, to identify hazardous road locations in Serbia. They compared and analyzed the strengths and weaknesses of each method, concluding that the objective data-based discrimination method yielded greater accuracy. Zahran et al. [3] developed a Spatial Traffic Accident Analysis (STAA) model, which considered accident frequency, severity, and social cost, to identify and rank accident black spots using historical accident data from a road section in Brunei. The results demonstrated that STAA outperformed other methods in terms of accuracy. Ferit et al. [4] applied the relative frequency method to establish the relationship between the number of accidents and the environmental characteristics of a road section based on accident data from an expressway in Turkey. This method proved to possess excellent detection ability and even demonstrated accurate identification for road sections that lacked accident data. However, historical accident data cannot be utilized for real-time risk identification and predicting potential accidents.

As a substitute for actual collisions, near-crash events are increasingly utilized in research related to driving risk. A near-crash event refers to a situation where the target vehicle or any other vehicle, pedestrian, bicycle, or animal needs to rapidly evade, satisfying the criteria of no collision, no premeditation, the need for avoidance, and the need for quick response [5]. These near-crash events provide controlled experimental data and serve as a valuable addition to real crash data in traffic safety research.

Advanced driver assistance systems (ADAS) help drivers with various driving tasks and capture motion parameters and real-time location data during driving. These motion parameters are directly related to traffic safety. By using historical data, a model can be developed to understand how motion parameters relate to the safety risk of a specific road section. This model, combined with real-time motion parameters, allows for real-time assessment of road section safety. Formos et al [6] conducted a study using traffic flow and vehicle-mounted data from the UK’s M1 highway. They developed a deep neural network (DNN) model that analyzed traffic conflicts by combining these data and calculating surrogate safety indicators (SSM). The model achieved 94% accuracy, showing that time-to-collision (TTC) varied with speed, weather, and traffic density. Gao et al [7] used the random forest algorithm and video trajectory data to identify potential collision risks during vehicle travel. Their multimodal deep convolutional neural network (DCNN) outperformed other models, achieving an AUC of 0.81. Xie et al [8] utilized networked driving data from Michigan to propose the time-to-disturbance collision (TTCD) as a method for capturing rear-end collision risks. Chen et al [9] introduced a collaborative driving scheme for Internet of Vehicles (IoV) intersections, demonstrating high accuracy in driver intent recognition, trajectory prediction, and collision probability assessment.

The analysis reveals that near-crash events can be utilized instead of historical accidents to comprehensively evaluate the road risk level in terms of potential accidents. There exists a potential connection between vehicle operating parameters and road safety level. ADAS-equipped vehicles can gather abundant near-crash events and operating parameters, enabling the establishment of a Bayesian network model to establish the relationship between them. Ultimately, by utilizing real-time vehicle operating parameters, the safety level of a road section can be evaluated.

Therefore, considering the current lack of methods that solely use a large amount of vehicle operation data for road risk assessment in existing traffic safety research, this study proposes a method based on connected Advanced Driver Assistance Systems (ADAS) to extract Near-crash events and classify road section risks. Road sections are first divided by type, and their risks are categorized into three severity levels (high, medium, and low) using Near-crash events extracted from connected vehicle driving data. A Bayesian network model is then established with Netica software to identify risks, supported by a road risk identification database constructed by matching driving data to each section. The main contributions of this paper are as follows:

1) Unlike traditional methods that rely on fixed monitoring devices such as microwave radars or video cameras for localized risk assessment, this study innovatively leverages collision warning information from connected vehicles’ ADAS systems to construct a dynamically updated road segment risk identification database. This approach enables comprehensive and real-time evaluation of traffic operation risks across the entire road network.
2) A Bayesian-based risk identification network is developed to analyze fundamental driving data such as speed, acceleration, and steering wheel angle, enabling real-time risk identification for each road segment. This method eliminates the need for costly roadside sensing infrastructure and can be seamlessly integrated into navigation apps, offering drivers real-time updates on road network risks and safe route planning suggestions.

## 2. Method

**Fig 1:**
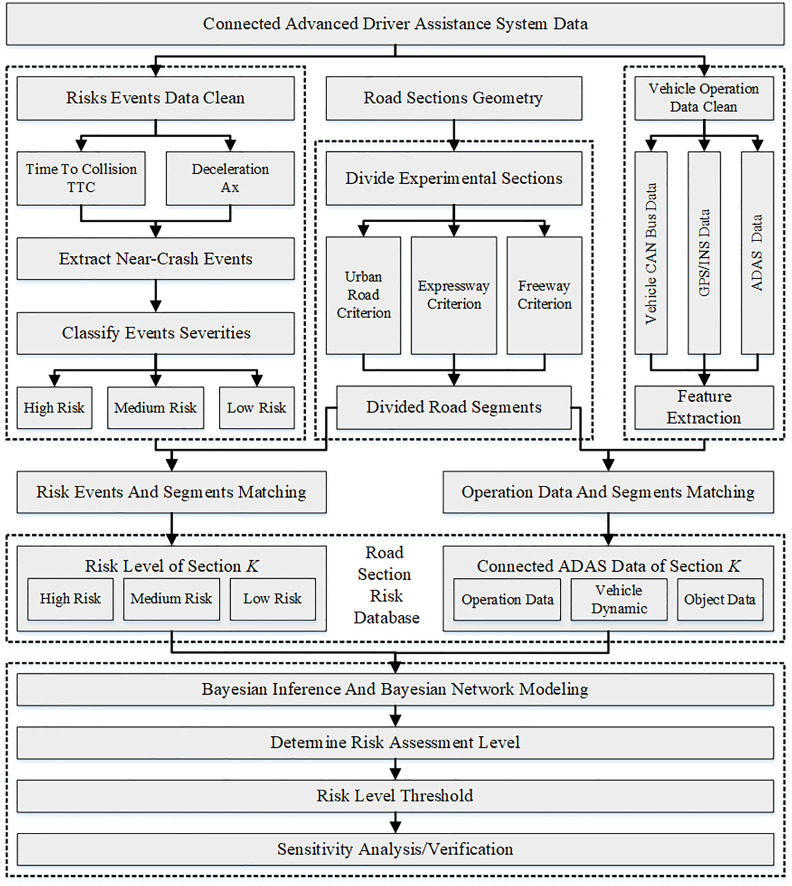
Research process.

The research methods of this paper are as follows. Firstly, road sections are categorized based on road type. Then, near-crash events are used to classify the risk of each section into high, medium, and low severity levels as output. Driving data is matched to each road section, creating a road risk identification database. A Bayesian network model is developed using Netica software to identify the risk of road sections, and its feasibility are verified. The research process is illustrated in Fig 1. The data used in this study was collected on public roads and is publicly available [10]. No specific permits or approvals were required from regulatory authorities, as the activities complied with local traffic laws. The data was used solely for research purposes, with no privacy concerns, and no ethical or legal issues arose during the process.

### 2.1 Road segment division

The data for this study is derived from real-world driving data collected from vehicles equipped with ADAS on urban roads, expressways, and freeways. Due to their variations in road layout, geometric design, and the arrangement of traffic signs and markings, as well as differences in driving behavior and types of risks involved, separate considerations are required for developing accident risk prediction models.

Road sections of different lengths have varying impacts on traffic conditions and subsequently affect the risk division. For instance, shorter road sections enable connected vehicles to more accurately reflect changes in traffic conditions, especially during congestion. However, they are susceptible to errors due to latitude, longitude, and GIS software limitations, resulting in poor stability and accuracy of vehicle data. Conversely, longer road sections provide more accurate and stable connected vehicle data, but only offer an average depiction of traffic conditions without accounting for the diffusion process during congestion. Therefore, the guiding principle for dividing road sections is to use the shortest length that still ensures accuracy and stability of the vehicle data index.

To divide urban expressways and freeways into sections, this study utilized the rule of equal intervals. However, when segmenting urban roads, adjacent intersections were treated as a single segment. This means that the latitude and longitude ($L_{n}$ , $B_{n}$) were gathered at the center point O of each intersection, and the actual distance between the two intersections determined the length of the road segment. The segments were then sequentially numbered as 1, 2...n, and the four corner points of each segment represented its range, as shown in the formula (1) and (2).


$$Road_{segment}=\{L_{1},L_{2},,L_{3},L_{4},\cdots L_{n}\}$$ (1)

$$L_{i_{scope}}=\{(lon_{i1},lat_{i1}),(lon_{i2},lat_{i2}),(lon_{i3},lat_{i3}),(lon_{i4},lat_{i4})\}$$ (2)

### 2.2 Classification method of road section risk level

#### 2.2.1 Extraction of near-crash events.

A near-crash event refers to a quick and dangerous evasive driving maneuver, such as sudden steering, braking, accelerating, or a combination of these actions [11]. Research [10] indicates that near-crash events are the most common type of collision replacement data. However, the definition of near-crash events differs among scholars, as they use different criteria to identify such events. Currently, near-crash events are primarily identified based on vehicle operating parameters and alternative safety indicators. Due to variations in these indicators and their threshold values across different studies, the number of near-crash events obtained from the same natural driving data can vary. Table 1 presents a summary of the methods employed by researchers to identify near-crash events.

**Table 1:** Definition of near-crash events.

Researchers	Methods
Guo et al [12]	Lateral acceleration; longitudinal acceleration; key events; forward collision time (TTC); rear collision time and yaw rate
Wang et al [13]	When the longitudinal acceleration reaches −1.5m/s2 and the lateral acceleration reaches -1m/s2, the event is considered a near-crash event
Naji et al [14]	The headway (THW) is less than 0.6s; the pressure of the brake booster is greater than 10Mpa; the braking acceleration is less than −0.4 m/s2; if one of these is satisfied, then it will be regarded as a near-crash event
Wu et al [15]	Post intrusion time (PET); proportion stop distance (PSD) and crash possible index (CPI)
Lyu et al [16]	THW is less than 0.5s; brake booster pressure is greater than 1.2Mpa; braking acceleration is less than −3 m/s2; if one of these is satisfied, then it is regarded as a near-crash event
Lyu et al [17]	When the longitudinal acceleration of the vehicle reaches -2m/s^2^, it is marked as the trigger point of the near-crash event, and the data from 15s before the trigger point to 5s after the trigger point is extracted

As shown in Table 1, Braking deceleration and Time to Collision (TTC) are commonly used predictors for driving risk. In the same lane, the group of front and rear vehicles can be considered as a driving and following group. The rear vehicle’s safe braking deceleration can be calculated based on the preceding vehicle’s emergency braking deceleration. This means that each vehicle’s deceleration reflects the driving risk of that vehicle [18]. TTC is the duration between the moment when two adjacent vehicles do not take any measures and a rear-end collision occurs [19].

In this study, collision time and braking deceleration were chosen as indicators to identify near-crash events. Relevant researchers [20–21] have suggested that TTC < 1.5 seconds or deceleration with longitudinal acceleration ax < −3.5 m/s² is an appropriate criterion for event extraction. Therefore, this study adopts a combined threshold of TTC < 1.5 s and ax < −3.5 m/s² as the standard for identifying near-crash events. If the interval between two events was less than 3 seconds, they were merged. Even if only one criterion was met, the event was still considered a near-crash event.

It is worth noting that TTC is commonly used to assess rear-end collision risks. Therefore, many scholars have developed various 2-D TTC models to evaluate multi-angle collision risks during lane-changing [21]. In this study, the TTC values are directly measured using the Mobileye advanced driver-assistance system. This system can simultaneously track up to eight targets ahead and output the TTC value with the highest collision probability during lane-changing [22].

#### 2.2.2 Quantification of road section risk levels.

Near-crash events have varying probabilities of progressing into actual collisions, with higher-risk events being more likely to result in collisions. Therefore, this study utilizes the extracted indicators of near-crash events, specifically braking deceleration and TTC, as parameters for clustering. The DBSCAN database in the Python clustering algorithm is employed to divide each near-crash event into three risk levels: high, medium, and low. These risk levels serve as the foundation for categorizing road sections based on their level of risk.

The more risk events occur on a stretch of road, the higher the level of risk events, the more dangerous the stretch. Based on this, this study calculated the risk score of the road section through the formula. x, y and z are the number of low, medium and high grade risk events respectively, and their weights are 1, 2 and 3 respectively.

The more the occurrence of risk events on a road segment, and the higher the level of those events, the more hazardous the segment becomes. This study calculates the risk score for each road section using the formula(3):


Score=x+2y+3z (3)

In the formula, x, y, and z represent the number of low, medium, and high-level risk events, respectively. The weights assigned to these events are 1, 2, and 3, respectively. Based on the calculated risk scores, the K-means clustering method was employed to classify the road segments into three risk levels: high, medium, and low.

### 2.3 Risk identification using the Bayesian network model

BN, commonly known as Bayesian Belief Network (BBN), possesses strong capabilities in addressing uncertain problems. It excels in learning and reasoning even when faced with limited and uncertain information. Furthermore, BN can effectively incorporate both prior knowledge and newly acquired information, allowing for the expression and fusion of multi-source information within the network. As a result, BN has found applications in various fields [23–24], including state detection, fault diagnosis, quality assessment, dynamic analysis, and risk management.

**Fig 2:**
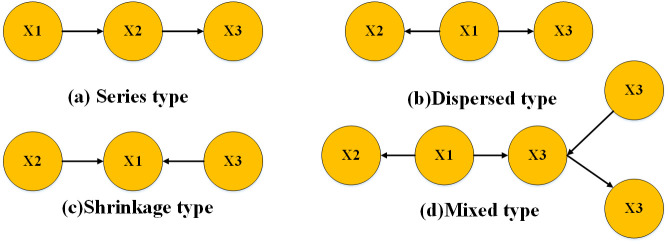
Four typical directed acyclic graphs.

Typically, Bayesian networks are represented using a directed acyclic graph (DAG) structure. This structure encompasses a set of random variables that capture their respective states and their probabilistic causal relationships. It is often denoted as $U=\left\{X_{1},X_{2},...,X_{n}\right\}$ Fig 2 presents commonly used DAG structures.

In Fig 2, $X_{1},X_{2},X_{3},X_{4},X_{5}$ represent variables with multiple states. The one-way arrows between the variables illustrate the causal relationships, with the direction indicating the relationship between the parent node and the child node. The child node is situated on the receiving side of the arrow. Four types of relationships exist between nodes, classified as series, dispersed, shrinkage, and mixed types. The mixed type can consist of any combination of the three aforementioned forms. In a DAG, the relationship between any set of child nodes and parent nodes can be determined by referring to the conditional probability table.

In a DAG, each node can be in either a true or false state. The conditional probability of $X_{1}$ depends on the true or false values of $X_{2}$ and $X_{3}$. Table 2 displays the conditional probability of $X_{1}$.

**Table 2:** Conditional probability of node $X_{1}$.

Conditional probability of $X_{1}$		$X_{2}$	
		T	F
$X_{3}$	T	$P_{11}$	$P_{12}$
	F	$P_{21}$	$P_{22}$

If a parent node in a DAG has no child nodes, then the prior probability of the state of the node can be calculated from the joint probability function of all variables in the DAG, which becomes a marginalized formula, as shown in Equations (4).


$$P(U)=P(X_{1},...,X_{n})=\prod_{i=1}^{n}P(X_{i}|pa(X_{i}))$$ (4)

Among them, $pa(X_{i})$ represents each parent node of node $X_{i}$. If there are relevant conditional independence assumptions, the above calculation can be further simplified.

The identification of road segment risk using a Bayesian network involves a structured process consisting of the following key steps:

- Step 1 Construction of the Directed Acyclic Graph-DAG: The first step is to establish the causal relationships between different nodes, which represent variables such as traffic conditions, driver behavior, and environmental factors. This involves defining the pointing relationships between nodes to construct a DAG, which serves as the foundational framework for the Bayesian network.
- Step 2 Calculation of Probabilities: Next, the prior probabilities of each node and the conditional probabilities between nodes are calculated. These probabilities are derived from historical data or expert knowledge.
- Step 3 Learning and Reasoning Process: Based on the prior probabilities of the evidence nodes (observed data) and the conditional probabilities between the evidence nodes and intermediate nodes, the probability values of the target node (road risk) are computed under different risk states. This step enables the network to infer the likelihood of risk based on the given evidence.
- Step 4 Risk Identification: Finally, leveraging the reasoning capabilities of the Bayesian network, driving data is used to evaluate and identify road risks.

## 3. Data preparation for risk identification modeling

### 3.1 Test route

**Fig 3:**
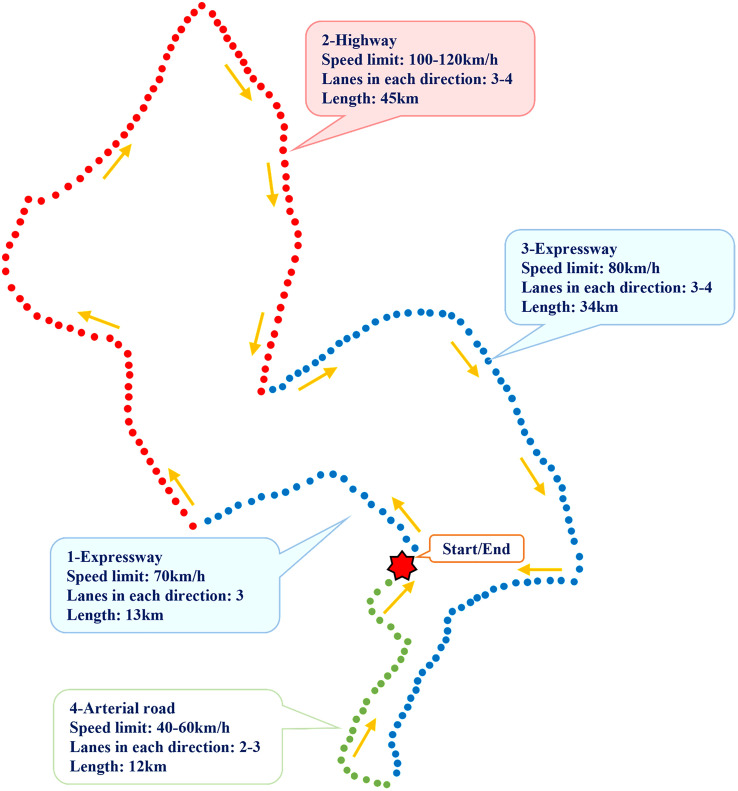
Natural driving test route.

To gather natural driving data from different types of roads, a 105 km mixed test route was chosen. The test route consists of a conventional urban road, two urban expressways, and a freeway. The specific route details can be found in Fig 3.

### 3.2 Onboard unit and connected driving data

To gather data on driver operating behavior, vehicle motion, driving environment, and surrounding vehicles during real-world driving, the research team developed an onboard unit. This unit incorporates various equipment, including the CAN bus, inertial navigation system, Mobileye, DVR, and other components. Table 3 provides a partial list of the obtained data. For this study, only the vehicle’s longitudinal acceleration from the inertial navigation system and the Time to Collision (TTC) from the Mobileye were utilized as indicators to identify near-crash events. Additionally, inputs to the Bayesian Network (BN) model included speed, lateral acceleration, longitudinal acceleration, yaw angle, accelerator, steering wheel angle, and steering wheel angle velocity.

**Table 3:** Data obtained from the natural driving test.

Data Sources	Variable	Definition	Unit
Vehicle CAN Bus	Accelerator Pedal (A_C_)	Control the engine throttle opening by controlling the amount of pedaling	%
	Brake Pressure	The force with which the car slows down or stops by braking	MPa
	Steering Angle*(A)*	The angle of rotation of the steering wheel	d
	Steering Angular Velocity*(A*_*v*_)	The angle of rotation of the steering wheel per second	d/s
	Vehicle Speed*(V)*	The distance traveled by the vehicle per unit time	Km/h
GPS/INS	Lateral Acceleration*(a*_*y*_)	Acceleration perpendicular to the direction of the vehicle	m/s^2^
	Longitudinal Acceleration(*a*_*x*_)	Acceleration along the direction of the car	m/s^2^
	Yaw Rate*(V*_*yr*_)	Deflection of the car about the vertical axis	d/s
	Longitude	The abscissa of the spherical coordinate system	d
	Latitude	The ordinate of the spherical coordinate system	d
ADAS	Distance from Right Lane Line	Distance between vehicle and right lane line	m
	Distance from Left Lane Line	Distance between vehicle and left lane line	m
	Distance Headway (*s*_*DHW*_)	In a platoon traveling in the same direction in one lane, the distance between the front and rear adjacent vehicles	m
	Time Headway (*t*_*THW*_)	The time difference between the front ends of two adjacent vehicles passing through the same place	s
	Time to Collision (*t*_*TTC*_)	The time when the car will collide with the car in front	s

### 3.3 Extraction of near-crash events

Based on the near-crash event definition standard described in 2.2, a total of 1,342 near-crash events were collected during the 10,000 km natural driving experiment. Using the Python DBSCAN database, these events were classified into three levels: high, medium, and low, based on the Time to Collision (TTC) and longitudinal acceleration. The clustering algorithm achieved a good result, as indicated by a silhouette coefficient $s_{\left(i\right)}$ of 0.84. Table 4 presents the statistics of the near-crash events at different risk levels: 205 high-risk events, 367 medium-risk events, and 770 low-risk events, following a pyramid-shaped distribution.

**Table 4:** Statistics of near-crash events at different levels.

Event Risk Level	Number of Events	Event Ratio	Mean And Standard Deviation of Events			
			*t*_*TTC*_		*a*_*x*_	
			Average	S.D.	Average	S.D.
High Level	205	15.35%	0.65	0.24	−1.34	1.22
Middle Level	367	27.35%	0.63	0.26	−1.17	1.28
Low Level	770	57.37%	1.04	0.20	−0.69	0.97

### 3.4 Risk classification of road sections

The experimental road was divided into 617 sections, which consisted of 124 urban road sections, 218 freeway sections, and 274 urban expressway sections. The average length of the road sections for each road type can be found in Table 5.

**Table 5:** The average length of the road sections for each road type.

Road Type	Freeways	Urban Expressways	Urban Roads
Average Length	200m	160m	116.7m

**Fig 4:**
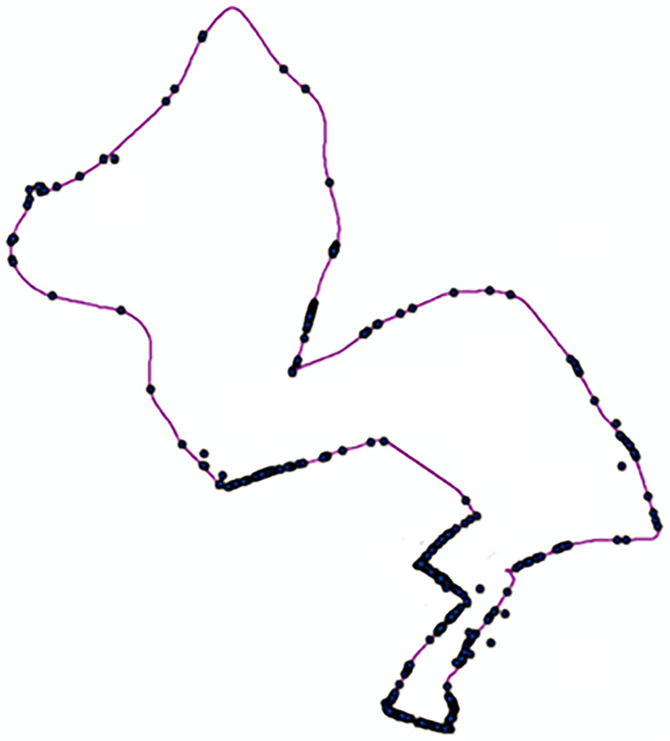
Risk points matched to road segments.

During the natural driving experiment, the vehicle recorded real-time latitude and longitude information. The nearest neighbor analysis function in ArcGIS was used to match the risk points to the divided road segments. First, the latitude and longitude of the risk points were converted from the geographic coordinate system to the projected coordinate system using the projection function in ArcGIS. Then, the projected risk points were matched to the route based on their x and y coordinates in the projected system. Examples of matching risk points to road segment information can be observed in Table 6, while the results of the matching process are depicted in Fig 4. Table 7 showcases the number of risk points for each risk level within each road segment and provides the total risk assessment score for each segment.

**Table 6:** Example of matching risk points to road segment information.

Numbering	Point Coordinates	Adjacent Road Section Number	Risk Point Level
1	(114.304,30.634)	577	Low
2	(114.304,30.633)	576	Low
3	(114.303,30.633)	576	Low
…	…	…	…

**Table 7:** The total risk score of each road segment.

Road Section	1	2	3	4	…	614	615	616	617
Low Level Number	0	2	11	2		0	0	0	0
Middle Level Number	1	0	10	0		0	1	0	0
High Level Number	0	0	6	0		0	0	0	0
Total Score	2	2	49	2		0	2	0	0

Using K-means clustering, the road segment scores were classified into three categories: high risk, medium risk, and low risk. The cluster center points for the three risk levels were 1.04, 29.84, and 101.25, respectively. Table 8 displays the number of road sections belonging to each risk level.

**Table 8:** Number of different types of road sections with different risk levels.

	Low Risk	Medium Risk	High Risk	Total
Urban Road	99	24	1	124
Urban Expressway	266	9	0	275
Freeway	211	4	3	218
Total	576	37	4	617

As shown in Table 8, the proportions of high risk, medium risk, and low risk sections in all three road types exhibit a pyramid distribution. This phenomenon aligns with the safety pyramid theory’s description of traffic event severity distribution [25]. Furthermore, as illustrated by the spatial distribution of risk points in Fig 3 and Fig 4, Freeway and Urban Expressway sections are longer, with risk points concentrated in a few critical locations. In contrast, risk points on Urban Roads are more evenly distributed across segment units, resulting in a higher proportion of medium-risk sections on Urban Roads.

### 3.5 Driving behavior database

For this research and analysis, the following indicators were selected: velocity, lateral acceleration, longitudinal acceleration, yaw rate, accelerator, steering angle, and steering angular velocity, as shown in Table 9. Real-time data collected during vehicle operation included speed, lateral acceleration, longitudinal acceleration, and yaw angular velocity, which were referred to as vehicle operating parameters. The accelerator, steering wheel angle, and steering angular velocity were associated with driver operating behaviors and referred to as vehicle control parameters. To match the driving data of all drivers with the road segments, latitude and longitude information was used, and the nearest neighbor analysis function in GIS was employed to assign each track point to its corresponding road segment.

**Table 9:** Driving behavior Indicator system.

Indicator Category	Indicator Name	Short Name	Unit
Vehicle Operating Parameters	Velocity	V	km/h
	Lateral Acceleration	*a*_*y*_	m/s^2^
	Longitudinal Acceleration	*a*_*x*_	m/s^2^
	Yaw Rate	*V*_*(yr)*_	d/s
Vehicle Controlling Parameters	Accelerator	*A*_*c*_	%
	Steering Angle	A	d
	Steering Angle Velocity	*V(a)*	d/s

Before constructing the BN model, it is important to establish the thresholds for these indicators. In this study, it is assumed that the majority (80%) of drivers’ behavior indicators are at the intermediate level, while a minority (20%) of drivers’ behavior indicators are at either the high or low level. With this assumption, the thresholds for each behavior indicator are determined using the 10th and 90th percentiles, resulting in three levels: High, Medium, and Low. The specific steps for determining these thresholds are as follows:

Step 1: Analyze the data on a certain road section by obtaining the 10% and 90% quantile data of the aforementioned indicators for each driver on a certain road section.

Step 2: Calculate the average value of these quantile values for all drivers on the same road section and use it as the percentile value of the final index for this road section.

Step 3: Take the average value obtained in step 2 on all the same road section types as the basis for the classification of the corresponding risk levels of the aforementioned indicators.

Following these steps, different indicators are divided into their respective high, medium, and low levels, as presented in Table 10.

**Table 10:** Value ranges corresponding to the high, medium, and low levels of different indicators.

Indicator	Indicator Range			Level
	Urban		Expressways	Freeways
V	V > 43.67	V > 65.43	V > 86.07	High
	38.00 < V < 43.67	62.55 < V < 65.43	83.41 < V < 86.07	Medium
	V < 38.00	V < 62.55	V < 83.41	Low
*a*_*y*_	|LAA|>0.23	|LAA|>0.36	|LAA|>0.33	High
	0.15<|LAA|<0.23	0.18<|LAA|<0.36	0.16<|LAA|<0.33	Medium
	|LAA|<0.15	|LAA|<0.18	|LAA|<0.16	Low
*a*_*x*_	|LOA|>0.60	|LOA|>0.30	|LOA|>0.27	High
	0.20<|LOA|<0.60	0.12<|LOA|<0.30	0.10<|LOA|<0.27	Medium
	|LOA|<0.2	|LOA|<0.12	|LOA|<0.10	Low
*V*_*(yr)*_	YA > 0.024	YA > 0.009	YA > 0.008	High
	0.010<|YA|<0.024	0.003<|YA|<0.009	0.003<|YA|<0.008	Medium
	|YA|<0.010	|YA|<0.003	|YA|<0.003	Low
*A*_*c*_	ACC > 19.85	ACC > 21.96	ACC > 26.13	High
	8.49 < ACC < 19.85	11.82 < ACC < 19.85	18.26 < ACC < 19.85	Medium
	ACC < 8.49	ACC < 11.82	ACC < 12.86	Low
A	|SWA|>5.30	|SWA|>2.00	|SWA|>1.97	High
	2.00<|SWA|<5.30	0.74<|SWA|<2.00	0.82<|SWA|<1.97	Medium
	|SWA|<2.00	|SWA|<0.74	|SWA|<0.82	Low
*V(a)*	SWAV>11.30	SWAV>5.40	SWAV>4.23	High
	2.46 < SWAV<11.30	0.95 < SWAV<5.40	0.62 < SWAV<4.23	Medium
	SWAV<2.46	SWAV<0.95	SWAV<0.62	Low

## 4. Road risk identification modeling

### 4.1 Construction of BN model

**Fig 5:**
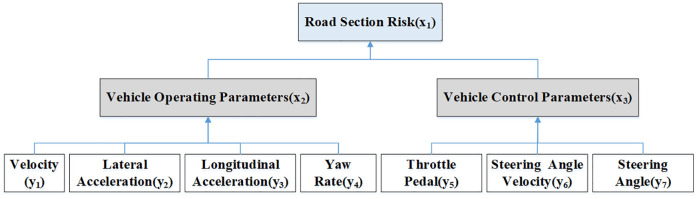
Structure of BN model for road segment risk identification.

Feature selection for the BN model was informed by recent developments in learning-based risk prediction models [26], we applied a triple BN model <V, E, ɵ> in this study, constructed based on the adopted indicators. E represents a finite set of edges, which depict one-to-one relationships between nodes. The value of ɵ reflects the degree of causal relationship among each node. V represents the node, with $V=(x_{i},y_{i})$ as a non-empty and finite set. $x_{i}$ denotes the hidden node in the network model, while $y_{i}$ represents the observation node. The specifics of each node are outlined in Table 11. The final constructed road segment risk BN structure model is illustrated in Fig 5.

**Table 11:** Specifics of vehicle operation risk nodes.

Node	Meaning	State
$x_{1}$	Road Risk Level	“Y”, “N”
$x_{2}$	Vehicle Operating Parameters	“Y”, “N”
$x_{3}$	Vehicle Control Parameters	“Y”, “N”
$y_{j}$	Impact Factor	——

### 4.2 Road risk identification model reasoning

#### 4.2.1 Model parameter establishment.

In the BN models for urban roads, expressways, and freeways, each node was in either a risk (Y) or non-risk (N) state, except for the observation nodes. The road section risk node’s risk state was represented by (R)={Y,N}, the vehicle operating parameters’ risk state was represented by (OR)={Y,N}, and the vehicle control parameters’ risk state by (CR)={Y,N}. The observation nodes included speed, lateral acceleration, longitudinal acceleration, yaw rate, accelerator, steering wheel angle, and steering wheel angle speed, with their states categorized as Low, Medium, or High. The probability and conditional probability for these states appearing in different road types are shown in Table 12.

**Table 12:** Occurrence probability and conditional probability of observation nodes in different road types.

Category	Index	Risk Level	Probability (%)			Conditional Probability (%)		
			Urban	Express	Highway	Urban	Express	Highway
Vehicle Operating Parameters	V (y_1_)	Low	40.7	44.9	47.8	41	59	56
		Medium	35.6	25.2	32.9	38	24	26
		High	23.7	29.9	19.3	21	17	18
	*a*_*y*_ (y_2_)	Low	49.2	44.6	52.9	39	52	45
		Medium	25.7	19.8	31.8	19	30	37
		High	25.1	35.6	15.3	42	18	18
	*a*_*x*_ (y_3_)	Low	50.2	54.7	54.0	47	49	55
		Medium	31.3	33.9	27.7	30	41	18
		High	18.5	11.4	18.3	23	10	27
	*V*_*(yr)*_ (y_4_)	Low	39.6	47.2	42.2	44	57	51
		Medium	31.9	28.9	30.1	26	28	21
		High	28.5	23.9	27.7	30	15	28
Vehicle Control Parameters	*A*_*c*_ (y_5_)	Low	45.6	37.8	49.7	35	39	48
		Medium	43.7	40.1	32.5	17	34	21
		High	10.7	22.1	17.8	48	27	29
	A (y_6_)	Low	44.2	42.9	51.8	28	53	48
		Medium	34.2	27.6	32.9	20	27	36
		High	21.6	29.5	15.3	52	20	16
	*V(a)* (y_7_)	Low	44.7	37.4	44.9	48	47	54
		Medium	38.2	42.1	29.9	23	30	27
		High	17.1	20.5	25.5	29	33	19

#### 4.2.2 Inference modeling based on Netica software.

**Fig 6:**
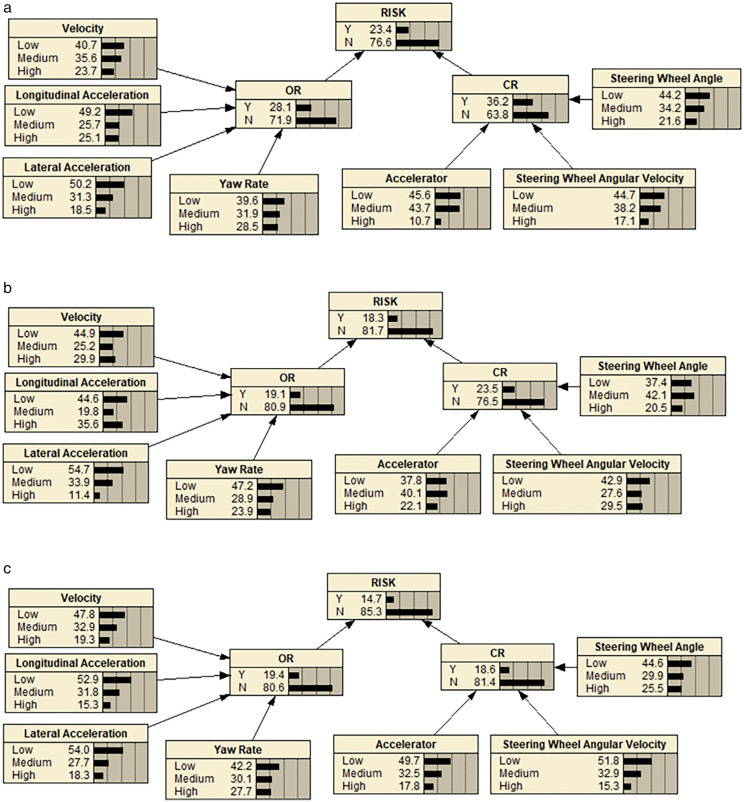
Three types of BN models for road segment risk. (a) BN model for risk of urban road sections. (b) BN model for risk of urban expressway sections. (c) BN model for risk of freeway sections].

We used Netica software to establish risk identification models. In order to do so, we needed to create a suitable BN model and input node data that is relevant to the analysis. This node data can be obtained from conditional probability tables that are based on expert assessments, or acquired through driving experiments with cyclic training. For our study, we chose the latter approach and used the “Equation” function to input the probability data. We then calculated the conditional probability of the hidden node by considering the correlation between different factors. The Fig 6 displays the proposed BN models for the three types of roads.

#### 4.2.3 Probability division of operational risk levels.

The risk levels were determined by the probability of risk occurrence, thus resulting in high, medium, and low risk. Table 13 illustrates the classification of road section operation risk levels.

**Table 13:** Classification of road section operation risk levels.

Risk Level	Risk Outcome	Risk Probability(%)
Level 1	High Risk	(75,100]
Level 2	Medium Risk	(50,75]
Level 3	Low Risk	(0,50]

### 4.3 Model sensitivity analysis

In the BN model, sensitivity analysis is a valuable tool for identifying the key factors that significantly impact the risk of road sections. Using the Netica software, we conducted sensitivity analysis on all nodes in the urban road sections BN model. The results are presented in Table 14. The analysis revealed that among all the observed nodes, Speed, lateral acceleration, and longitudinal acceleration had the greatest influence on the operational risk of urban road sections. For expressways and freeways, we used the same approach to determine the three observed nodes that have the most significant impact on the risk of road sections.

**Table 14:** Sensitivity analysis of driving risk on urban road sections.

Node	Interactive Information	Sensitivity to R
R	0.98	100
OR	0.25	37.4
CR	0.16	21.9
V	1.3×10^−2^	9.77
LAA	1.0×10^−2^	5.64
LOA	8.2×10^−3^	3.03
AC	5.9×10^−3^	1.44
SWA	2.9×10^−3^	0.60
YA	1.2×10^−3^	0.20
SWAV	9.7×10^−4^	0.13

Next, we moved forward with altering the parameter value of the three observed nodes that have the highest impact on the risk of road sections. The sensitivity of the models to these changes was assessed by observing how they affected the final risk probability. Sensitivity analysis was performed on all three models.

**Fig 7:**
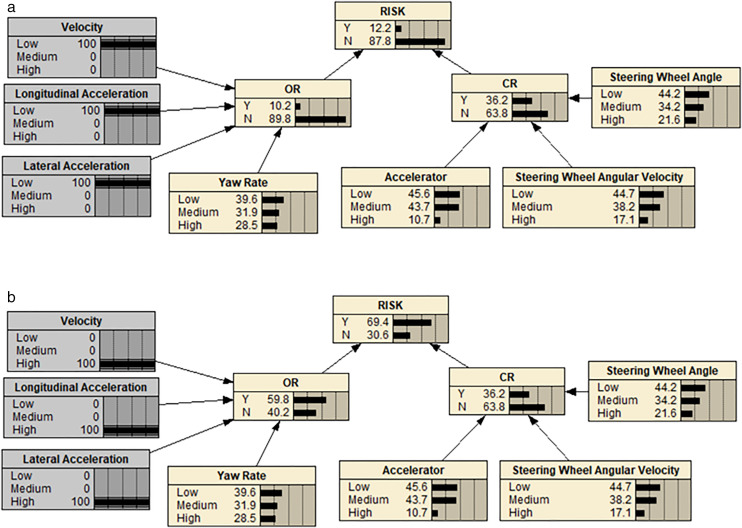
BN model when the risk index of urban road sections change. (a) When the risk index of urban road sections decreased. (b) When the risk index of urban road sections increased.

Assuming the speed, lateral acceleration, and longitudinal acceleration were set at 100% in the low-risk segment, the BN deduction showed a decrease in the operational risk level from 23.4% to 12.2%, as seen in Fig 7(a). Conversely, assuming the same settings in the high-risk segment, the BN deduction indicated an increase in the operational risk level from 23.4% to 69.5%, as depicted in Fig 7(b). By altering the speed, lateral acceleration, and longitudinal acceleration, it was observed that the risk probability of urban road operations experienced significant fluctuations, demonstrating the sensitivity of the proposed model.

**Fig 8:**
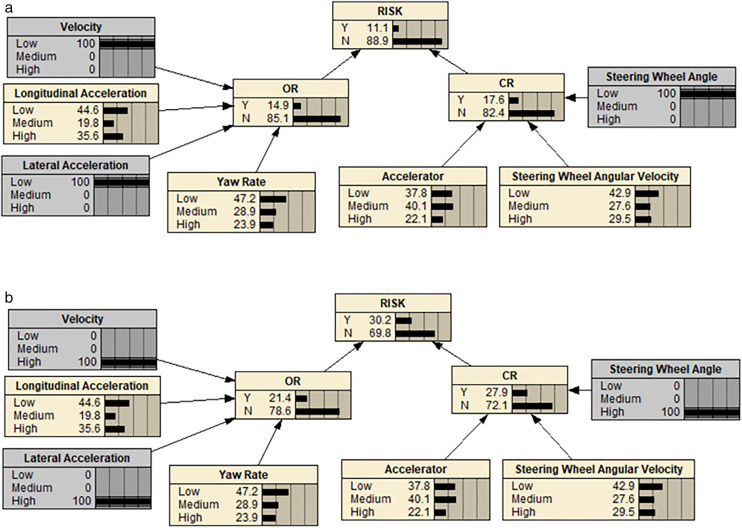
BN model when the risk index of urban expressway sections increased. (a) When the risk index of urban expressway sections decreased. (b) When the risk index of urban expressway sections increased.

Lateral acceleration, speed, and steering angle were found to have the greatest impact on the operational risk of urban expressway sections. Assuming these factors were set at 100% in the low-risk segment, the BN deduction revealed a decrease in the operation risk level from 18.3% to 11.1%, as shown in Fig 8(a). Conversely, assuming the same settings in the high-risk segment, the BN deduction indicated an increase in the operation risk level from 18.3% to 30.2%, as illustrated in Fig 8(b).

**Fig 9:**
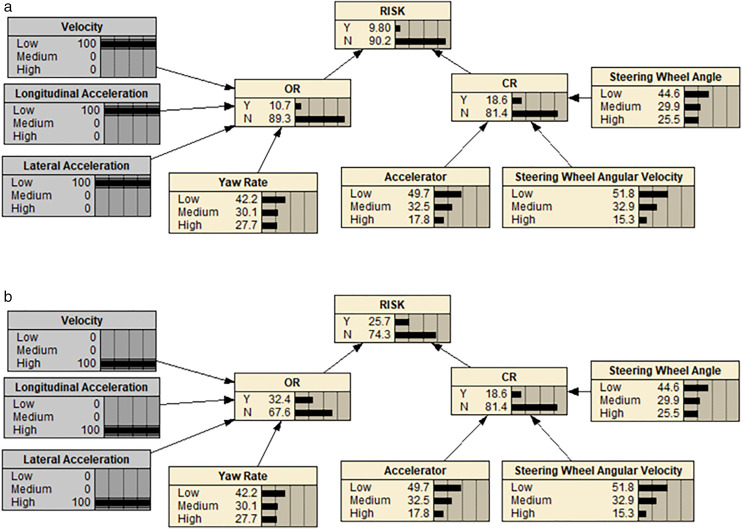
BN model when the risk index of freeway sections increased. (a) When the risk index of freeway sections decreased. (b) When the risk index of freeway sections increased.

For freeways, longitudinal acceleration, speed, and lateral acceleration were found to have the greatest impact on freeway operation risk. Assuming these factors were set at 100% in the low-risk segment, the BN deduction revealed a decrease in the operation risk level from 14.7% to 9.8%, as shown in Fig 9(a). On the other hand, assuming the same settings in the high-risk segment, the BN deduction indicated an increase in the operation risk level from 14.7% to 25.7%, as illustrated in Fig 9(b).

The changes in operation risk for the mentioned road sections are summarized in Table 15. It can be observed that as the risk probability of a specific sensitive factor of a road section significantly increased or decreased, the corresponding risk level of that section also underwent significant change. This demonstrates the strong sensitivity of the proposed BN models for urban road sections, urban expressway sections, and freeway sections.

**Table 15:** Influence of Observed Node Changes on Model Results.

	Urban Roads	Expressway	Freeway
Normal Road Segment Risk	23.4%	18.3%	14.7%
Risk of Sensitive Factors Decreased	12.2%	11.1%	9.8%
Risk of Sensitive Factors Increased	69.5%	30.2%	25.7%

## 5 Conclusion

This study utilizes near-crash events to determine risk levels of road sections and establishes a Bayesian network (BN) model to capture the relationship between driving behavior and road section risk levels. The main conclusions and contributions of this study are as follows:

(1) This study proposes a method for extracting near – crash events and identifying road section risks using connected ADAS. Analyzing 10,000 km of natural driving test data, we extracted 205 high-risk, 367 medium-risk, and 770 low-risk events, laying a data-driven foundation for subsequent analyses.
(2) A novel road section risk level classification method is developed. We divided three road types (urban roads, urban expressways, and freeways) into sections. By matching near – crash events of different levels to road sections based on driving-collected longitude and latitude data and calculating risks according to event frequencies, we identified 576 low-risk, 37 medium-risk, and 4 high-risk sections. This method provides a practical approach for quantifying road risks.
(3) Employing Netica software, we established separate BN models for different road types to explore the link between driving behavior and road section risk. These models demonstrated high sensitivity.

This study has several limitations. First, due to the challenges in obtaining accurate accident data, near-crash events were used as a proxy for real crash data, which may not fully capture the complexities of actual collisions. Second, our analysis is based on basic ADAS with risk warning functions. While sufficient for our research, these basic systems may lack the advanced capabilities of more sophisticated ADAS. Recent advancements like Automatic Emergency Braking (AEB), Lane Departure Warning (LDW), Lane Keeping Assistance (LKA), or Blind Spot Warning (BSW) [27–28] can provide more information related to potential vehicle collision risks, thus enhancing the accuracy and applicability of our risk assessment model.

Future work will focus on integrating advanced ADAS systems to enhance risk identification precision and analyzing data from moments preceding near-crash events to optimize model performance under complex traffic conditions. Additionally, statistical methods will be explored to quantify structural differences among BN models across road types, investigating the influence of road design versus driving behavior patterns. These efforts aim to improve the robustness and applicability of traffic safety solutions.
